# L-Selectin/CD62L Is a Key Driver of Non-Alcoholic Steatohepatitis in Mice and Men

**DOI:** 10.3390/cells9051106

**Published:** 2020-04-29

**Authors:** Hannah K. Drescher, Angela Schippers, Stefanie Rosenhain, Felix Gremse, Laura Bongiovanni, Alain de Bruin, Sreepradha Eswaran, Suchira U. Gallage, Dominik Pfister, Marta Szydlowska, Mathias Heikenwalder, Sabine Weiskirchen, Norbert Wagner, Christian Trautwein, Ralf Weiskirchen, Daniela C. Kroy

**Affiliations:** 1 Department of Internal Medicine III, University Hospital, RWTH Aachen, 52074 Aachen, Germany; ctrautwein@ukaachen.de (C.T.); danielakroy@gmail.com (D.C.K.); 2 Division of Gastroenterology, Massachusetts General Hospital and Harvard Medical School, Boston, MA 02114, USA; 3 Department of Pediatrics, University Hospital, RWTH Aachen, 52074 Aachen, Germany; anschippers@ukaachen.de (A.S.); seswaran@ukaachen.de (S.E.); nwagner@ukaachen.de (N.W.); 4 Institute for Experimental Molecular Imaging, University Hospital, RWTH Aachen University, 52074 Aachen, Germany; srosenhain@ukaachen.de (S.R.); fgremse@ukaachen.de (F.G.); 5 Dutch Molecular Pathology Centre, Department of Pathobiology, Faculty of Veterinary Medicine, Utrecht University, 3508 Utrecht, The Netherlands; l.bongiovanni@uu.nl (L.B.); a.debruin@uu.nl (A.d.B.); 6 Division of Chronic Inflammation and Cancer, German Cancer Research Center Heidelberg (DKFZ), 69120 Heidelberg, Germany; suchira.gallage@med.uni-heidelberg.de (S.U.G.); dominik.pfister@med.uni-heidelberg.de (D.P.); marta.szydlowska@med.uni-heidelberg.de (M.S.); mathias.heikenwaelder@med.uni-heidelberg.de (M.H.); 7 Institute of Molecular Pathobiochemistry, Experimental Gene Therapy and Clinical Chemistry (IFMPEGKC), University Hospital, RWTH Aachen University, 52074 Aachen, Germany; sweiskirchen@ukaachen.de (S.W.); rweiskirchen@ukaachen.de (R.W.)

**Keywords:** non-alcoholic steatohepatitis, NASH, CD62L, L-Selectin, insulin resistance

## Abstract

CD62L (L-Selectin) dependent lymphocyte infiltration is known to induce inflammatory bowel disease (IBD), while its function in the liver, especially in non-alcoholic steatohepatitis (NASH), remains unclear. We here investigated the functional role of CD62L in NASH in humans as well as in two mouse models of steatohepatitis. Hepatic expression of a soluble form of CD62L (sCD62L) was measured in patients with steatosis and NASH. Furthermore, CD62L^−/−^ mice were fed with a methionine and choline deficient (MCD) diet for 4 weeks or with a high fat diet (HFD) for 24 weeks. Patients with NASH displayed increased serum levels of sCD62L. Hepatic CD62L expression was higher in patients with steatosis and increased dramatically in NASH patients. Interestingly, compared to wild type (WT) mice, MCD and HFD-treated CD62L^−/−^ mice were protected from diet-induced steatohepatitis. This was reflected by less fat accumulation in hepatocytes and a dampened manifestation of the metabolic syndrome with an improved insulin resistance and decreased cholesterol and triglyceride levels. Consistent with ameliorated disease, CD62L^−/−^ animals exhibited an enhanced hepatic infiltration of Treg cells and a strong activation of an anti-oxidative stress response. Those changes finally resulted in less fibrosis in CD62L^−/−^ mice. Additionally, this effect could be reproduced in a therapeutic setting by administrating an anti-CD62L blocking antibody. CD62L expression in humans and mice correlates with disease activity of steatohepatitis. CD62L knockout and anti-CD62L-treated mice are protected from diet-induced steatohepatitis suggesting that CD62L is a promising target for therapeutic interventions in NASH.

## 1. Introduction

One of the most common chronic and progressive liver diseases in Western countries that develops in the absence of alcohol abuse is non-alcoholic fatty liver disease (NAFLD). With rising prevalence, NAFLD is expected to be evolving to the leading cause for liver transplantation and recognized as a major health burden [1,2,3]. The typical disease course ranges from simple steatosis, easily evolving to more advanced hepatic alterations, such as non-alcoholic steatohepatitis (NASH), concomitant with fibrosis and cirrhosis; finally resulting in the development of end-stage liver disease, such as hepatocellular carcinoma (HCC) [4,5].

The growing medical problem gets evident when considering recent studies estimating that the prevalence of NAFLD cases associated with adult obesity will increase by 21% until 2030, NASH even by 63%. Moreover, the prevalence for NAFLD in adults is more than 30% of the total population with around 20% being diagnosed as NASH. Looking at these developments, calculations show that the incidence of HCC and NASH-related liver transplantation will be doubled until 2030 [3,6,7,8,9,10]. Often described as hepatic manifestation of the metabolic syndrome, NASH is accompanied by obesity, abdominal fat deposition, type 2 diabetes, insulin resistance, and dyslipidaemia [11]. Common pathological alterations on the molecular level include an impaired lipid metabolism, together with hepatocellular accumulation of fatty acids provoking mitochondrial dysfunction, hepatocyte ballooning, and cell death leading to immune cell infiltration, oxidative stress and, finally, fibrosis [12,13].

Clinical trials show promising results in therapeutically targeting NASH [14,15,16]. However, at present there is no effective approved pharmacological treatment [10,17]. Consequently, it is crucial to better define the molecular mechanisms leading to NASH development and progression.

Immune cell infiltration into inflamed tissue is a highly regulated and rigorously controlled multistep process involving different cell adhesion molecules [18,19,20]. L-Selectin (CD62L) is a member of the selectin family of adhesion molecules and mediates the initial attachment and subsequent rolling of leukocytes on activated endothelium [21,22,23]. It predominantly binds to inducible ligands on the endothelium at the site of inflammation, whereas the mucosal addressin cell-adhesion molecule-1 (MAdCAM-1) is the most abundant interaction partner [19,24,25].

CD62L is expressed on leukocytes, especially on neutrophils, monocytes and most types of lymphocytes [26,27]. On neutrophils, CD62L plays a special role as it is constitutively expressed at high levels and has an important impact on their chemotaxis, recruitment and activation [28,29,30]. After neutrophil activation and migration, CD62L expression is rapidly decreased by shedding from the cell surface by ADAM-17 (a disintegrin and metalloproteinase metallopeptidase domain 17) [31]. This shedding and the release of the soluble form of CD62L (sCD62L) further regulates leukocyte recruitment and immune cell reactivity [32,33,34]. Appropriately, different studies show elevated serum levels of sCD62L in patients with systemic lupus erythematosus [35] and chronic lung disease [36]. In mice, the blockade of CD62L results in decreased neutrophil infiltration accompanied by reduced liver enzymes in a liver ischemia reperfusion model, suggesting a crucial role for CD62L in liver diseases [37].

In recent years, selective inhibition of different adhesion molecules to block immune cell infiltration in chronic inflammatory diseases such as inflammatory bowel disease (IBD) reached clinical application [38]. Since during chronic inflammation adhesion molecule-dependent infiltration of immune cells to the liver is an important step in the progression from NAFLD to NASH, the aim of this study was to analyse the expression of CD62L in human samples from patients with acute and chronic liver injury. Further, we investigated the effects of CD62L inhibition in two different murine diet-induced steatohepatitis models (high fat diet (HFD) and methionine and choline deficient (MCD)). We thereby demonstrate that CD62L expression is altered in human NAFLD/NASH and that deletion and therapeutic blockade of CD62L in mice prevents development and progression of steatohepatitis by mediating immune cell infiltration and thereby reducing the hepatic oxidative stress environment. Hence, CD62L seems to be a promising therapeutic target in NAFLD and NASH.

## 2. Materials and Methods

### 2.1. Human Samples

Liver specimens were obtained according to the local Ethics committee rules from liver explants or liver resection recruited at the RWTH Aachen University Hospital. The study, as presented, was approved by the Institutional Review Board under permit number EK166-12. When intrahepatic lesions were present, the tissue for this study was collected from the most distant section of the specimen. For reverse transcription quantitative real-time PCR (RT-qPCR), we analysed 12 samples with steatosis and NASH. Flow cytometric analysis of intrahepatic immune cells was performed from 3 control patients and 5 NASH patients.

### 2.2. Animal Studies

#### 2.2.1. Animal Welfare

The study was carried out in accordance with Animal Research: Reporting of In Vivo Experiments (ARRIVE) guidelines and the law of the regional authorities for the Nature, Environment and Consumer Protection of North Rhine-Westphalia (LANUV, Recklinghausen, Germany) and approved by the respective Committee (Permit Number: 84-02.04.2014.A010). All experiments were performed in accordance with the German guidelines for animal housing and husbandry. Treatments were in accordance with the criteria of the German administrative panel on laboratory animal care. To prevent cage effects, we performed weekly bedding exchange between cages. Health reports of the animals are available in Supplementary material.

#### 2.2.2. Housing and Generation of Mice

Male C57BL/6J wild type mice (WT) and male CD62L-deficient mice (CD62L^−/−^) all on C57BL/6J background were used as experimental animals. At least 8 chow fed animals, 8 MCD fed animals, and 8 HFD fed animals per group and time point were treated in parallel. For antibody treatment, 4 animals per group were treated in parallel. Respective WT and CD62L^−/−^ animals were age-matched and always littermates. All experiments were repeated at least twice in independent experimental setups. Animals were housed in the animal facility of the University Hospital RWTH Aachen with 12-h light/dark cycles and water and food ad libitum available.

#### 2.2.3. Dietary Treatments

Dietary treatments were performed with 12-week-old male mice weighing at least 25 g. Mice were fed chow, methionine choline deficient diet (MCD) (Sniff, Soest, Germany; cat. no. E15653-94) or high fat diet (HFD) (40 kcal % fat (vegetable fats), 20 kcal % fructose, 2% cholesterol) (Brogaarden, Lynge, Denmark; cat. no. D09100301). WT and CD62L^−/−^ mice showed a food intake of about 6 g per mouse per day without any differences between groups.

#### 2.2.4. Therapeutic Antibody Treatments

Steatohepatitis was induced in WT animals by feeding MCD for 4 weeks. For additional 2 weeks, mice were fed MCD and in parallel treated with 4 µg/g body weight CD62L blocking antibody or isotype control (BioLegend, San Diego, CA, USA; cat. no. 104402, cat. no. 400544) by intraperitoneal injections every other day.

#### 2.2.5. Glucose and Pyruvat Tolerance Test

Animals were fasted for 6 h and blood glucose was measured via taking one drop of blood from the animal tails after cutting the very tip using an Accu-Check® Aviva meter (Roche, Basel, Switzerland) every 15 min following intraperitoneal administration of 2 g/kg glucose or 0.2 g/kg pyruvate for 2 h.

#### 2.2.6. μ Computer Tomography (μCT) Imaging

In vivo μCT imaging of WT and knockout (KO) mice fed with HFD (*n* = 6) for 24 weeks was performed using an ultra-low-dose, high throughput, flat-panel, in vivo X-ray microcomputed tomography scanner (SkyScan 1278, Bruker, Kontich, Belgium). The X-ray tubes of the μCT were operated at a voltage of 59 kV with a current of 831 uA. To cover the entire mouse, a continuous rotation scan was performed with one full rotation (360°) in 0.569 (deg) rotation steps, exposure time of 39ms, total scan duration of 51s, and dose estimation of 79 mGy. Animals were anaesthetized using 2% isoflurane in air for the entire imaging protocol (flow rate 1 L min^−1^). After acquisition, volumetric data sets were reconstructed using a modified Feldkamp algorithm with a smooth kernel at an isotropic voxel size of 207 μm. The fat-containing tissue regions, which appear hypo-intense in the μCT data, were segmented using an automated segmentation method with interactive correction of segmentation errors using the Imalytics Preclinical software (Gremse-IT GmbH, Aachen, Germany) [39]. The volumetric fat percentage was computed as the ratio of (subcutaneous and visceral) fat volume to the entire body volume.

#### 2.2.7. Blood Collection

Blood from mice was collected by retro-orbital bleeding. Therefore, mice were anaesthetised with isoflurane inhalation and blood was collected via a glass capillary. Samples were aliquoted and serum was stored at −80 °C.

### 2.3. Human CD62L Serum Elisa

The 10 µL serum samples of patients were analysed from 6 control patients, 26 NASH, and 10 NAFLD with the human sL-Selectin/CD62L ELISA kit (R&D Systems, Minneapolis, MI, cat. no DY728) in accordance with the manufacturer’s instructions. The measurement of sCD62L levels in serum via ELISA was performed in 54 serum samples. These samples were obtained from 6 control patients, 26 NASH, and 10 NAFLD patients.

### 2.4. AST/ALT Analysis

Serum aspartate aminotransferase (AST) and serum alanine aminotransferase (ALT) levels were measured by the Central Laboratory Facility of the University Hospital, RWTH Aachen.

### 2.5. NAFLD Activity Score

Histopathological scoring of liver sections and their validation was performed by Professor Dr. Alain de Bruin at the University Utrecht via a NAFLD activity score (NAS), as described previously [40,41].

### 2.6. Insulin Measurement/Homeostatic Model Assessment for Insulin Resistance (HOMA-IR) Calculation

For measuring serum insulin levels, mice were fasted for 6 h and serum insulin was measured via the Ultra-Sensitive Mouse Insulin Elisa Kit (Crystal Chem, Zaandam, Netherlands) in accordance with the manufacturer’s instructions. The HOMA-Insulin resistance was calculated by correlation of the fasted plasma glucose and serum insulin levels.

### 2.7. Hepatic Triglycerides

The measurement of hepatic triglycerides was performed in 20 mg liver tissue, which was homogenized in 1 mL of a homogenization buffer (10 mM Tris, 2 mM EDTA, 0.25 M sucrose, pH 7.5). The standard curve was prepared in accordance with the manufacturer’s instructions of the Instruchemie LiquiColor mono Kit (Instruchemie, Delfzijl, Netherlands). Moreover, 200 µL of the kit reagent were added to 2 µL of each sample to the standard solution and incubated for 45 min at room temperature. After that, the optical density (OD) was measured at 492 nm within 15 min.

### 2.8. Hepatic Free Fatty Acids and Hepatic Cholesterol

For intrahepatic free fatty acids and cholesterol quantification the total amount of lipids within 20 mg snap frozen liver tissue were extracted with methanol-chloroform extraction. The concentration of either free fatty acids (FFA) or cholesterol was measured with the FFA quantification kit (Abcam, Cambridge, UK; cat. no. ab65341) and the cholesterol determination kit (Sigma-Aldrich, St. Louis, MO, USA; cat. no. MAK043) in accordance with the manufacturer’s instructions.

### 2.9. Hydroxyproline

The content of the collagen specific amino acid hydroxyproline was measured to quantify liver fibrosis. Therefore, colorimetric analysis of hydroxyproline in 20 mg snap frozen liver tissue was performed.

### 2.10. Histology, Sirius Red, and Oil Red O Staining

For haematoxylin and eosin (HE) stain, liver tissue samples were fixed in 4% formaldehyde, embedded in paraffin, cut and stained with HE. Pictures were taken using an Axio-Imager Z1 (Carl Zeiss, Jena, Germany) for each time point and genotype. Sirius Red staining was performed on paraffin liver sections. Slides were incubated in a solution containing 0.1% Sirius Red and 0.1% picric acid (pH 2.0) for 1 h. After that, incubation with 0.1 M HCl for 5 min was followed by treatment with an ascending ethanol series and finally incubated in Roti-Histol (Roth, Karlsruhe, Germany) and covered with Roti-Histokit (Roth, Karlsruhe, Germany). For analysis, 10 photographs per liver slide were analysed under polarised light in a 400× magnification. The Sirius Red positive areas were analysed via colour error measurement using the open source software Image-J. The intensity of the light reflection from the collagen filaments was morphometrically measured and used for the calculation. Oil Red O staining was performed on liver tissue samples snap frozen in OCT compound (optimal cutting temperature embedding medium for frozen tissue specimens). After fixation with 4% formalin, slides were washed with PBS and then stained with Oil Red O staining solution (Sigma-Adrich, Steindorf, Germany). After rinsing with water, nuclei were counter-stained with haematoxylin.

### 2.11. Immunofluorescence Staining

Liver tissue samples were snap frozen in OCT compound and were cut in 5-mm sections. After air-drying for at least 30 min, samples were fixed with ice-cold 4% paraformaldehyde. Primary antibodies (Table 1) were incubated in 1% mouse serum and 0.02% sodium acetat (Sigma Aldrich, Taufkirchen, Germany) in PBS (PAA, Vienna, Austria) for 1h at room temperature. Secondary antibodies for detection were AlexaFluor 488–conjugated or Alexa Fluor 594-conjugated (Molecular Probes, Boston, MA, USA). Nuclei were counterstained with DAPI (Vector Laboratories/Axxora, Loerrach, Germany). The detection of immunofluorescent signals was performed with an AxioImager Z1 microscope (Carl Zeiss, Jena, Germany). Moreover, 5 images at a 400× magnification per tissue slide of at least 6 animals per group and time point were counted.

### 2.12. Gene Expression Analysis by Real-Time PCR

Total mRNA was isolated from snap frozen liver tissue samples using the peqGOLD RNAPure™ kit (Peqlab, Erlangen, Germany) in accordance to the manufacturer’s recommendations. cDNA synthesis was performed of 500 ng mRNA after DNAse I digestion with a DNAse I kit (Invitrogen, Karlsruhe, Germany). Subsequently reverse transcription with the Omniscript reverse-transcription kit (Qiagen, Hilden, Germany) according to the manufacturer’s instructions was performed and used in a real-time PCR assay (Applied Biosystems, Foster City, CA, USA). cDNA expression was detected for specific genes of interest. Therefore, the SybrGreen tqPCR Supermix (Invitrogen) was used. All primers were obtained from MWG Biotech (Ebersberg, Germany). Primer sequences for human and murine primers can be found in Table 2. Analysis was performed as recommend by the manufacturer.

### 2.13. Isolation of Cells and Flow Cytometry

Intrahepatic leukocytes from whole liver extracts were isolated. Liver were perfused with 10 mL phosphate-buffered saline (PBS), cut up with scissors, and digested with collagenase IV (Worthington) at 37 °C for 30 min. Digested livers were filtered through a 70 µm cell strainer and intrahepatic leukocytes were stained for 20 min at 4 °C with fluorochrome-labelled monoclonal antibodies (Table 3). Then, cells were subjected to flow cytometry using a BD Canto II (BD Biosciences, Heidelberg, Germany). Data were analysed using FlowJo software (TreeStar, Ashland, OR, USA).

### 2.14. Isolation of Primary Liver Sinusoidal Endothelial Cells

Primary liver sinusoidal endothelial cells were isolated by perfusing the liver with 25 mL isolation buffer SC1, 25 mL SC2 with separately added pronase E (50 mg/150 mL), and 25 mL SC2 with separately added collagenase IV (100 mg/150 mL). After an additional 20 min incubation in SC2 with pronase E (50 mg/150 mL), collagenase IV (50 mg/150 mL), and DNAse I (150 µL/10 mL) at 37 °C the cell suspension was filtered through a 70 µm cell strainer and centrifuged for 1 min with 50× *g* at 4 °C. Supernatant was centrifuged (400× *g*, 8 min, 4 °C) and washed twice with Gey’s balanced salt solution (GBSS)-B containing DNAse I (150 µL/10 mL). Density gradient centrifugation was performed by dissolving the pellet in 34 mL GBSS-B and adding 14 mL Nycodenz I solution (5.18 g Nycodenz in 15 mL GBSS-A. For preparing the gradient, 12 mL Nycodenz II (3,63 g Nycodenz in GBSS-A) was filled in a tube and overlayed with 24.9 mL cell suspension and 3 mL GBSS-B solution, prior to centrifugation for 20 min with 500× *g* at 4 °C without a break. The second layer contains Kupffer cells and sinusoidal endothelial cells, which can be separated by plating for 20 min on a plastic dish, whereby, Kupffer cells get adherent after 20 min, while endothelial cells stay in the supernatant. The composition of used buffers is depicted in Table 4.

### 2.15. Cell Culture Experiments with Primary Liver Sinusoidal Endothelial Cells and Hepa1-6 Cells

Primary liver sinusoidal endothelial cells were isolated. Prior to cytokine stimulation cells were starved in Dulbecco’s modified eagle medium (DMEM) without fetal calf serum (FCS) (starving medium) for 16 h and stimulated with bovine serum albumin (BSA) as control or tumour necrosis factor-α (TNF-α) (25 ng/mL) for 24 h. The supernatant further termed conditioned medium (CM) was harvested and Hepa1-6 cells (derived from BW7756 tumour in a C57L mouse, Sigma-Aldrich) were stimulated with CM of BSA (control), CM of TNF-α stimulated liver sinusoidal endothelial cells, and additional 300 µM linoleic acid-oleic acid-albumin (OA+LA) or OA+LA alone for 24 h.

### 2.16. SDS PAGE and Western Blot

Snap frozen liver tissue was lysed in ice cold lysis buffer (1 M NaCl, 0.01 M EGTA, 0.5 M EDTA, 1 M NaH_2_PO_4_, 1 M Tris (pH 7.5), 1 M NaF, 10% Triton, 0.1 M PMSF, 1 mM Na_3_VO_4_), and protein lysate was heat denaturated (5 min, 95 °C) in double-strength sodium dodecyl sulphate sample buffer containing dithiothreitol before resolution in 10% SDS-PAGE. Primary antibody incubation of the membrane was substantiated with anti-α-mooth muscle actin (α-SMA) (ab32575, Abcam, Cambridge, UK), anti-Collagen1α1 (600-401-103, Rockland, Gilbertsville, PA, USA) and anti-GAPDH (MCA4739, AbD Serotec, Hercules, CA, USA) antibodies. For detection, the secondary antibodies HRP-linked anti-rabbit immunoglobulin G (7074S, Cell Signaling, Frankfurt, Germany) and HRP-linked anti-mouse immunoglobulin G (sc-2005, Santa Cruz Biotech., Santa Cruz, CA, USA) were used. Visualization of the antigen-antibody complexes were detected using the enhanced chemiluminescence (ECL) Chemiluminescence Kit (GE Healthcare, Buckinghamshire, UK). Densitometric analysis were performed with ImageJ software (U.S. National Institutes of Health, Bethesda, MA, USA) to measure the density of each protein band.

### 2.17. Quantification and Statistics

All numerical results are expressed as mean ± SEM and represent data from at least 6 animals per time point. Calculations via manual counting of immunostained cells were done with five to 10 high power fields/liver at a magnification of 40×. All significant p values were measured using the Student’s t-test when comparing two groups or ANOVA testing with Tukey’s multiple comparison post-test. Values of *p* < 0.05 were considered significant (* *p* < 0.05, ** *p* < 0.01, and *** *p* < 0.001).

## 3. Results

### 3.1. L-Selectin/CD62L is Increased in Patients With Acute and Chronic Liver Injury

To determine the significance of CD62L in patients with NAFLD and NASH, we measured serum levels of the soluble form of CD62L (sCD62L) in control patients, patients with NASH and NAFLD patients. Clinical and demographic characteristics of patients are summarized in Table S1.

**Figure 1:**
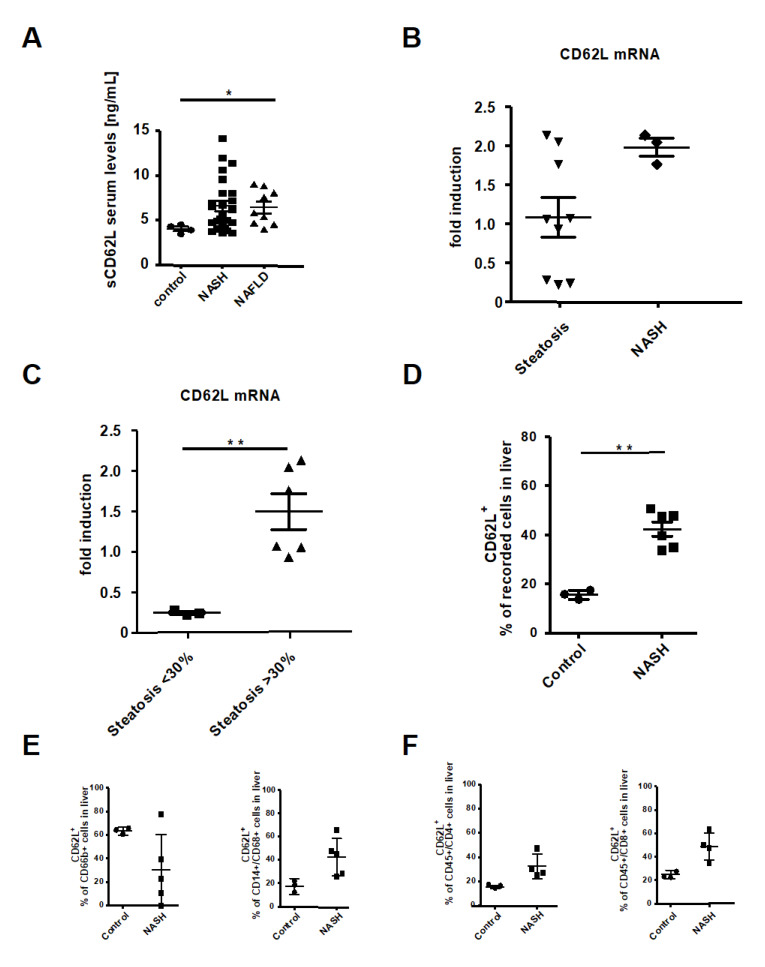
CD62L (L-Selectin) is increased in non-alcoholic steatohepatitis (NASH) patients. (**A**) Serum levels of soluble L-Selectin were measured via ELISA in depicted patient groups (* *p* < 0.05). (**B**) Liver biopsy samples were analysed for CD62L mRNA expression by RT-qPCR. The values were expressed as fold induction over the mean values obtained for control patient liver biopsies. (**C**) Results from (**B**) were grouped by patients with <30% and <30% steatosis. Values were expressed as fold induction over the mean value obtained for control patient samples (** *p* < 0.01). (**D**) Intrahepatic CD62L^+^ cells were analysed by flow cytometry in depicted patient groups. Cells were gated via, forward scatter/side scatter (FSC/SSC) duplets were excluded, living cells, CD45^+^/CD62L^+^. Depicted is the analysis of the percentage of recorded cells (* *p* < 0.05, ** *p* < 0.01). (**E**) Intrahepatic CD62^+^/CD66b^+^ neutrophils and CD14^+^/CD68^+^/CD62L^+^ monocytes were analysed by FACS. Analysis included control patients and patients suffering from NASH. Intrahepatic cells were gated by FSC/SSC, duplets were excluded, living cells, CD45^+^, CD66b^+^, CD62L or CD14^+^/CD68^+^, CD62L^+^. Displayed is the percentage of CD62L^+^ cells in liver biopsy samples. (**F**) Intrahepatic CD4^+^/CD62L^+^ and CD8^+^/CD62L^+^ T cells were analysed by FACS in control and NASH patients. CD4^+^/CD62L^+^ and CD8^+^/CD62L^+^ T cells were gated by FSC/SSC, duplets were excluded, living cells, CD45^+^, CD4^+^/CD62L^+^ or CD8^+^/CD62L^+^. Results are displayed as percentage of CD62L^+^ cells (** *p* < 0.01).

Patients with NAFLD and NASH showed increased serum levels of sCD62L compared to controls (Figure 1A). To investigate the expression of CD62L in NASH development and progression in more detail, we next evaluated the intrahepatic CD62L expression on mRNA level in steatosis and NASH patients. NASH patients exhibited a trend to more CD62L mRNA expression compared to Steatosis patients (Figure 1B). Steatosis patients were further sub grouped into patients with <30% liver fat and patients with >30% liver fat based on the liver steatosis grades introduced by Qayyum et al. (mild, 5–30% fat, moderate to severe >34%) [42]. Subgroup analysis of steatosis patients clearly revealed a direct correlation of fat accumulating within the liver >30% with an increase in intrahepatic CD62L mRNA expression compared to patients with a liver fat <30% (Figure 1C). Interestingly, there was an increased expression of β7-Integrin in steatosis patients compared to NASH and control which showed a significant upregulation in patients with <30% fat compared to those with >30% (Figure S1A).

MAdCAM-1 is one of the endothelial receptors for CD62L and β7-Integrin together with the intercellular adhesion molecule-1 (ICAM-1) and vascular adhesion molecule-1 (VCAM-1). MAdCAM-1 showed a comparable expression pattern than CD62L with significantly lower expression in steatosis patients compared to the NASH group, especially when the amount of fat is <30% (Figure S1B–D). To find the source of intrahepatic CD62L expression, flow cytometric analysis of the intrahepatic immune cell infiltrate was performed and revealed a significant increase in the number of CD62L^+^ cells in patients with NASH compared to controls (Figure 1D). Deeper analysis of CD62L^+^ immune cell subpopulations interestingly showed no significant differences in the percentage of CD62L^+^ neutrophils between groups (Figure 1E). In comparison to control samples, the increase in CD62L expression on immune cells in patients with NASH was found in CD14^+^CD68^+^ monocytes, CD4^+^ and CD8^+^ T cells (Figure 1E,F).

### 3.2. Deletion of CD62L Improves Metabolic Disorders and Steatosis in Diet-Induced Steatohepatitis

**Figure 2:**
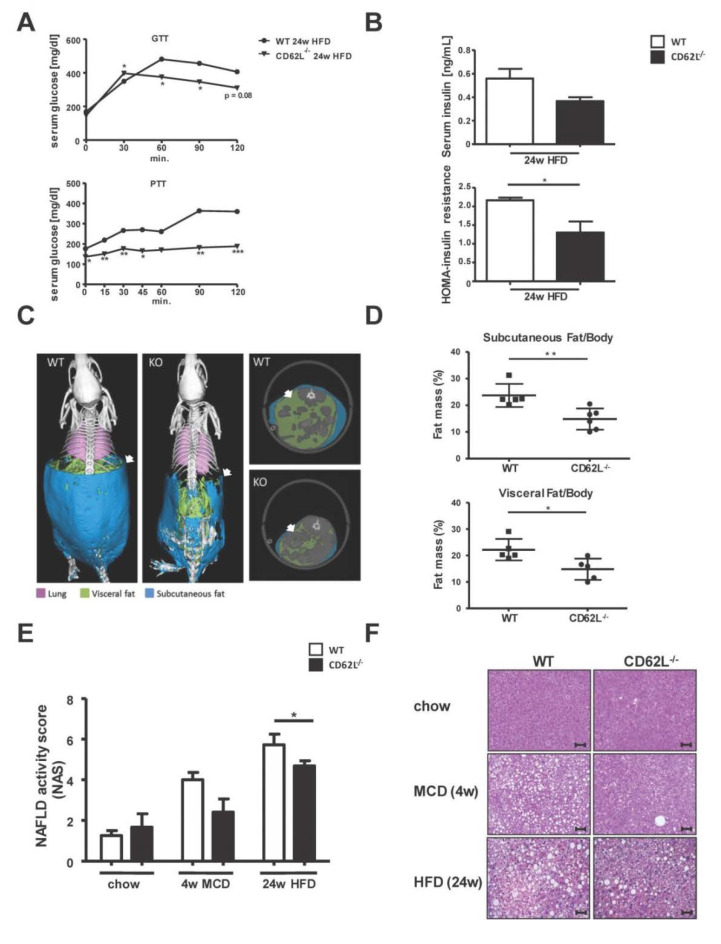
CD62L^−/−^ mice show less fat accumulation, steatosis, and metabolic changes during high fat diet (HFD) feeding. (**A**) Glucose (GTT) and pyruvate (PTT) tolerance test after 24 weeks HFD feeding in wild type (WT) and CD62L^−/−^ mice. Animals were fasted for 6 h and blood glucose was monitored following intraperitoneal injection of either 2.0 g/kg glucose or 2.0 g/kg pyruvate (* *p* < 0.05, ** *p* < 0.01, *** *p* < 0.001). (**B**) Insulin levels in serum of WT and CD62L^−/−^animals after 24 weeks HFD treatment. The calculation of the HOMA-insulin resistance is based on plasma glucose and serum insulin levels (*n* = 8), (* *p* < 0.05). (**C**) Three-dimensional volume renderings of segmented bones (white), lungs (pink), visceral fat (green), and subcutaneous fat (blue) upon in vivo μCT imaging of WT and CD62L^−/−^ mice after 24 weeks HFD feeding and 2D cross-sectional μCT images in transversal planes of the abdomen of mice. (**D**) Quantification of subcutaneous and visceral fat tissue (*n* = 6) of WT mice after 24 weeks of HFD. All data are expressed as mean ± SEM. Differences between WT and KO mice were determined using an unpaired, two-tailed t-test (* *p* < 0.05, ** *p* < 0.01). (**E**) Lower non-alcoholic fatty liver disease (NAFLD) activity score in CD62L^−/−^ animals compared to WT mice after 4 w methionine and choline deficient (MCD) and 24 w HFD treatment. The used NAS considers steatosis, lobular inflammation and hepatocellular ballooning. The histopathological validation was performed by two board-certified veterinary pathologists (L.B. and A.B.). (**F**) Representative images of hematoxylin & eosin (H&E) stained livers of WT and CD62L^−/−^ mice after chow, 4 w MCD or 24 w HFD feeding (400×, scale bars 50 µm, *n* = 8). All experiments were repeated at least twice.

To mechanistically determine the role of CD62L in NASH development and subsequent progression, we fed 12-week-old WT and CD62L^−/−^ mice with steatosis and steatohepatitis inducing diets: MCD for 4 weeks and HFD for 24 weeks. The total bodyweight changes comparably in WT and CD62L^−/−^ mice during 4 weeks of MCD treatment (Figure S2A). During the course of HFD feeding CD62L^−/−^ mice gain less weight while an equal food consumption of about 6g per mouse per day could be observed (Figure S2B). After 24 weeks of HFD feeding, animals with CD62L deficiency exhibited significantly less pronounced features of the metabolic syndrome compared to equally treated WT mice. This was demonstrated by faster dropping glucose levels after glucose injection and even no raise of serum glucose after pyruvate administration (Figure 2A). CD62L^−/−^ mice further displayed lower serum insulin levels and significantly decreased insulin resistance as evidenced by the HOMA-score (Figure 2B).

Next, subcutaneous and visceral fat accumulation was measured by µCT analysis. Here CD62L^−/−^ mice compared to WT controls showed significantly lower subcutaneous and visceral fat accumulation after 24 weeks HFD feeding (Figure 2C,D).

Longitudinal measurement of the fat ratio (fat volume/body volume) after 16 and 24 weeks HFD showed a decrease in the overall fat ratio in WT (Figure S3A–D) and, to a lesser extent, in CD62L^−/−^ animals (Figure S4A–D).

In parallel, histological analysis was performed using H&E stain that revealed an increase in hepatocyte vacuolization with more ballooned hepatocytes and micro- and macrosteatosis after MCD (4 weeks) and HFD (24 weeks) feeding. This observation was less pronounced in CD62L^−/−^ mice with a lower NAFLD activity score (NAS) in CD62L^−/−^ animals (Figure 2E, Figure S5), fewer hepatocyte ballooning, and just the beginning of fine fat droplet formation (Figure 2F).

Along with less disease progression in CD62L^−/−^ mice, a decreased liver to body weight ratio was found after HFD feeding (Figure S6A). Molecular analysis of the fat composition in serum and liver tissue revealed significantly decreased levels of serum, intrahepatic triglycerides (Figure S6B), and hepatic free fatty acids (Figure S6C) in CD62L^−/−^ animals after MCD and HFD compared to WT. At the same time, no changes in the hepatic cholesterol content were obvious (Figure S6D). Oil Red O stain showed that reduced steatosis development is further associated with hepatocyte vacuolization and less changes in liver architecture in CD62L^−/−^ mice (Figure S6E).

### 3.3. CD62L Deficiency Strongly Activates the Anti-Inflammatory Immune Response

**Figure 3:**
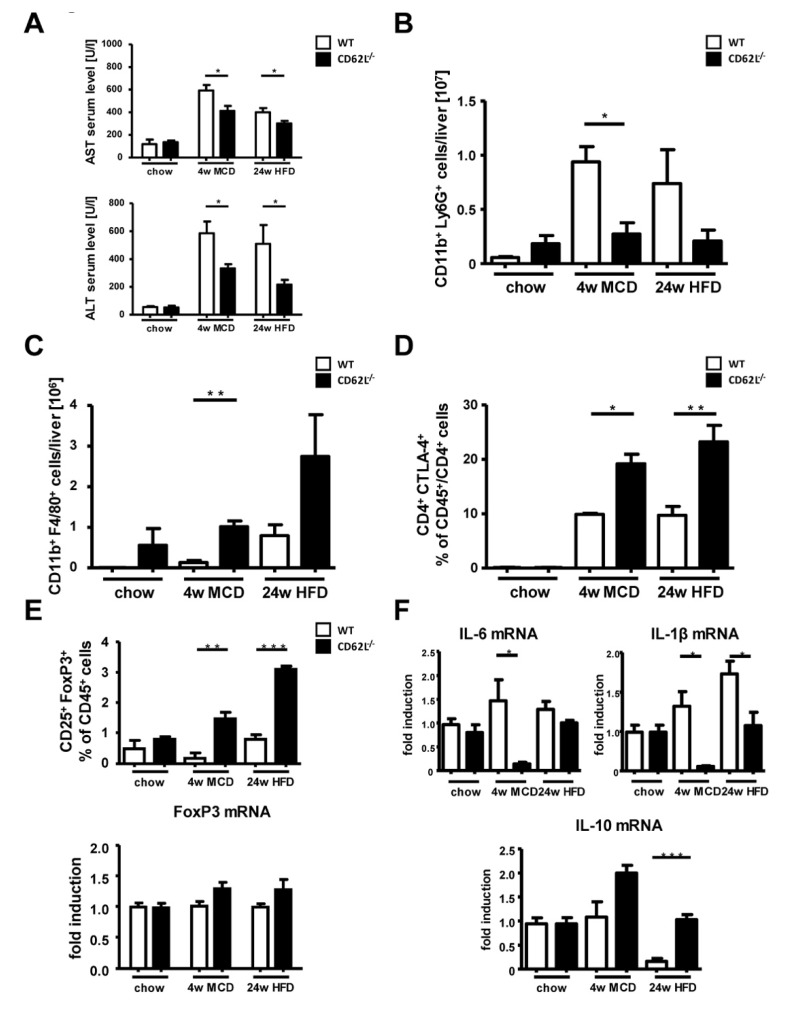
Stronger anti-inflammatory changes in the composition of immune modulatory cells in CD62L^−/−^ mice during steatohepatitis-inducing diets. (**A**) Serum aspartate aminotransferase (AST) and alanine aminotransferase (ALT) levels of wild type (WT) and CD62L^−/−^ animals after chow, 4 weeks MCD or 24 weeks HFD treatment. (* *p* < 0.05). (**B**) Total numbers of intrahepatic neutrophils were analysed by FACS. Cells were gated by FSC/SSC; duplets were excluded. Live/CD45^+^, CD11b^+^/Ly6G^+^ were regarded as neutrophils (* *p* < 0.05). (**C**) Total cell numbers of intrahepatic CD11b^+^/F4/80^+^ cells were analysed by FACS. Cells were gated by FSC/SSC; duplets were excluded. Live/CD45^+^, then gated on CD11b^+^ and F4/80^+^ (** *p* < 0.01). (**D**) Intrahepatic CD4^+^/CTLA4^+^ T cells were analysed by FACS. CD4^+^/CTLA4^+^ T cells were gated by FSC/SSC, duplets were excluded, Live/CD45^+^, CD4^+^/CTLA4^+^. A statistical analysis of the amount of CD4^+^/CTLA4^+^ cells of recorded CD45^+^ cells was performed. (* *p* < 0.05, ** *p* < 0.01). (**E**) Intrahepatic CD25^+^/FoxP3^+^ cells were analysed by FACS. Cells were gated by FSC/SSC, duplets were excluded, Live/CD45^+^, CD4^+^, CD25^+^/FoxP3^+^. A statistical analysis of the percentage of CD25^+^/FoxP3^+^ of recorded CD45^+^ cells was performed. Whole livers of chow, MCD and HFD fed WT and CD62L^−/−^ mice were analysed for FoxP3 mRNA expression via RT-qPCR (** *p* < 0.01, *** *p* < 0,001) and values expressed as fold induction over the mean values obtained for chow fed WT liver tissue. (**F**) Whole liver homogenates of chow, MCD and HFD fed WT and CD62L^−/−^ mice were analysed for IL-6, IL-1β and IL-10 mRNA expression via RT-qPCR and values expressed as fold induction over the mean values obtained for WT liver tissue (* *p* < 0.05, ** *p* < 0.01). All data are expressed as mean ± SEM (*n* = 8). All experiments were repeated at least twice.

Improvement in liver histology in CD62L^−/−^ mice after MCD and HFD challenge was reflected by significantly lower serum transaminases in CD62L^−/−^ animals compared to WT (Figure 3A). Hepatic inflammation by intrahepatic immune cell infiltration is one of the most characteristic factors to differentiate between simple steatosis and developing steatohepatitis. Flow cytometric analysis (FACS) was performed to determine hepatic immune cell infiltration in WT and CD62L^−/−^ mice after chow, MCD (4 weeks), and HFD (24 weeks) feeding. FACS revealed significant differences in the myeloid derived immune cell compartment with a decrease in CD11b^+^ Ly6G^+^ neutrophil infiltration (Figure 3B, Figure S7A) and a simultaneous rise in liver infiltration of CD11b+ F4/80+ macrophages in CD62L^−/−^ mice (Figure 3C, Figure S7B).

The percentage of CD4 and CD8^+^ T cells was not significantly different between WT and CD62L^−/−^ at all treatment conditions (Figure S6C,D). However, we characterized CD4+ immune cell subpopulations in more detail. Collectively, the CD4^+^ T cell fraction showed a stronger immune-regulatory activity by a significant increase in the number of CD4^+^CTLA4^+^ T cells in CD62L^−/−^ mice after MCD and HFD feeding (Figure 3D). Supporting the findings of a stronger anti-inflammatory immune response in CD62L^−/−^ mice, we found that this observation is further strengthened by a significant increase in the number of CD25^+^FoxP3^+^ regulatory T cells (Treg) corroborated by a slight trend to higher expression of FoxP3 (Forkhead box P3) on mRNA level (Figure 3E). To better understand the differences in immune regulation in CD62L^−/−^ and WT animals, we performed RT-qPCR analysis of the typical pro-inflammatory markers Interleukin-6 (IL-6) and IL-1β which were down regulated in CD62L^−/−^ mice compared to WT controls after MCD and HFD (Figure 3F). These alterations were not seen when mice were fed a regular chow diet, suggesting that an additional trigger inducing steatosis is necessary to increase respective inflammatory marker genes.

Ameliorated disease progression in CD62L^−/−^ animals was further reflected by a significant upregulation of the anti-inflammatory marker IL-10 after both steatohepatitis inducing diets (Figure 3F). Thus, CD62L deficiency lead to an enhanced anti-inflammatory immune response in the HFD model, but also in the MCD model of steatohepatitis, which is in agreement with a milder phenotype during NASH progression.

### 3.4. Less Steatohepatitis Progression in CD62L^−/−^ Mice is Associated with Reduced Oxidative Stress Response

Oxidative stress and the dampened activation of the anti-oxidative stress response has been described as the crucial factor involved in NASH development and progression [43,44]. To assess the oxidative status and the capacity of the anti-oxidative stress response in experimentally induced NASH, we analysed the production of superoxide as an indicator for oxidative stress within the liver tissue by immunofluorescent staining for Dihydroethidium (DHE) (Figure S8A). Quantitative evaluation of the amount of reactive oxygen species (ROS) superoxide by DHE+ cells revealed a strong imbalance of the hepatic oxidative metabolism with a significant shift towards a strong oxidative stress reaction in livers of WT animals after MCD and HFD treatment whereas CD62L^−/−^ animals showed no ROS production (Figure S8B). Moreover, we detected a significant increase of the anti-oxidative stress response in MCD and HFD treated CD62L^−/−^ mice compared to WT, indicated by a significant up-regulation of nuclear factor erythroid 2-related factor 2 (Nrf2) and Heme oxygenase-1 (HO-1) mRNA (Figure S8C,D). The strong activation of the anti-oxidative stress response goes along with the previous results showing amelioration of diet-induced steatohepatitis in CD62L^−/−^ animals, and may at least, in part, be responsible for ameliorated disease initiation and progression.

### 3.5. Deletion of CD62L Does Not Lead to Compensatory Upregulation of Adhesion Molecules in the Liver

We tested if loss of CD62L leads to compensatory expression of related cell adhesion molecules and, thus, performed immunofluorescence staining for other homing/receptor interaction partners. Vascular cell adhesion molecule-1 (VCAM-1) (Figure S9A,B) and intercellular adhesion molecule-1 (ICAM-1) (Figure S9C,D) showed a marked rise in WT animals when treated with steatohepatitis inducing diets. However, this upregulation was not observed in CD62L^−/−^ mice. In addition, the mRNA expression of the metalloprotease ADAM-17, a family member of the ADAM protein family, which serves as disintegrin for CD62L41, was not changed in WT mice during disease (Figure S9E), whereas its expression even dropped in CD62L^−/−^ animals.

### 3.6. Loss of CD62L Reduces Fibrosis Progression after MCD and HFD Feeding

**Figure 4:**
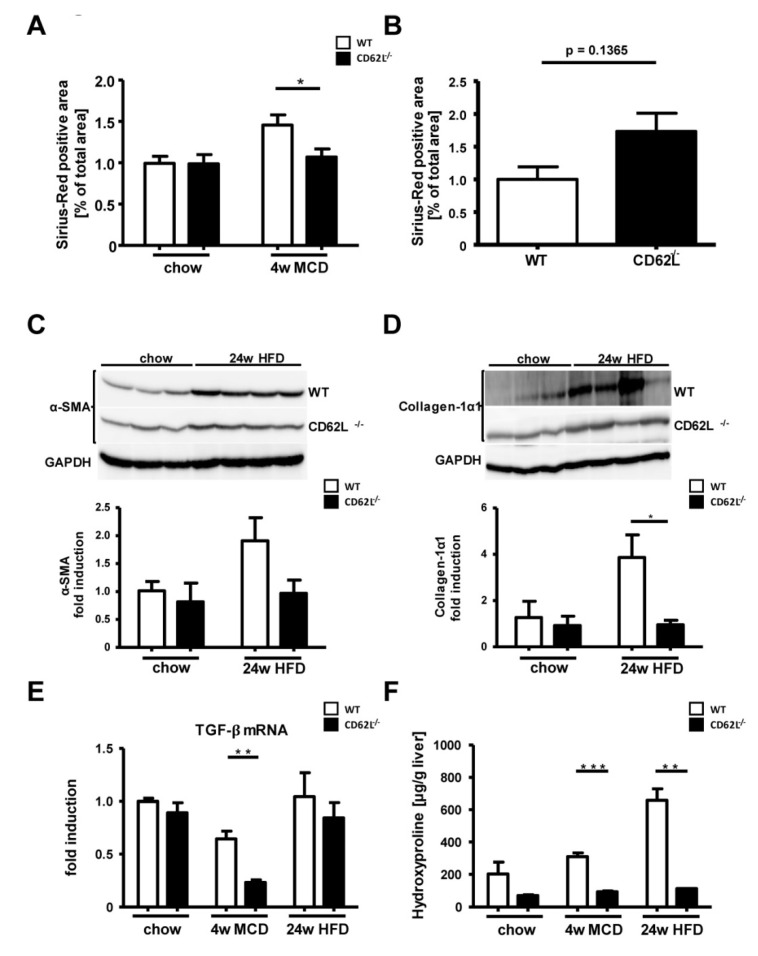
Weaker onset of fibrosis in CD62L^−/−^ mice in two models of dietary induced steatohepatitis. (**A**) Quantitative analysis of Sirius Red staining of liver sections of chow and 4 w MCD fed WT and CD62L^−/−^ mice was performed by calculating the Sirius Red positive area per view field of 10 view fields/liver by ImageJ (* *p* < 0.05). (**B**) Quantitative analysis of Sirius Red staining of liver sections of WT and CD62L^−/−^ mice after 24 w HFD feeding was performed by calculating the Sirius Red positive area per slide. (**C**) α-SMA Western blot analysis from liver extracts of WT and CD62L^−/−^ animals. (**D**) Collagen 1α1 Western blot analysis of liver extracts of chow and HFD fed WT and CD62L^−/−^ animals (* *p* < 0.05). (**E**) Whole liver homogenates of chow, MCD (4 w) and HFD (24 w) fed WT and CD62L^−/−^ mice were analysed for TGF-β mRNA expression via RT-qPCR. Values are expressed as fold induction over the mean values obtained for WT liver tissue (** *p* < 0.01). (**F**) Intrahepatic hydroxyproline content of WT and CD62L^−/−^ animals after treatment with either chow diet or steatohepatitis-inducing diets (4 w MCD, 24 w HFD) (** *p* < 0.01, *** *p* < 0.001). All data are expressed as mean ± SEM (*n* = 8). All experiments were repeated at least twice.

Activation of hepatic stellate cells together with excessive extracellular matrix (ECM) production triggers NASH progression towards fibrosis. Fibrosis progression in MCD and HFD fed WT and CD62L^−/−^ mice was first assessed by Sirius Red staining (Figure S10). Within liver tissue of WT animals, a slight increase in ECM accumulation was observed after 4 weeks of MCD treatment, which is absent in animals deficient for CD62L (Figure 4A). Moreover, 24 weeks of HFD showed in CD62L^−/−^ mice a weak increase Sirius Red positive area. However, there was a strong variation between animals, not leading to a significant difference (Figure 4B). In order to investigate this difference in collagen deposition in more detail, we performed Western blot analysis for α-SMA (Figure 4C) and Collagen-1α1 (Figure 4D). In contrast to CD62L^−/−^ mice, WT animals displayed increased levels of α-SMA and Collagen-1α1 after 24 weeks of HFD feeding. Moreover, liver tissue of CD62L^−/−^ animals exhibited less transforming growth factor-β (TGF-β) mRNA expression (Figure 4E) and significant reduction in hydroxyproline levels (Figure 4F) compared to liver tissue of MCD- or HFD-treated WT mice. These results indicate a less severe activation of hepatic stellate cells with decreased collagen deposition and fibrosis initiation in CD62L^−/−^ livers.

### 3.7. CD62L Deficiency Leads to Increased Activation of the Anti-Oxidative Stress Response in Hepatocytes

**Figure 5:**
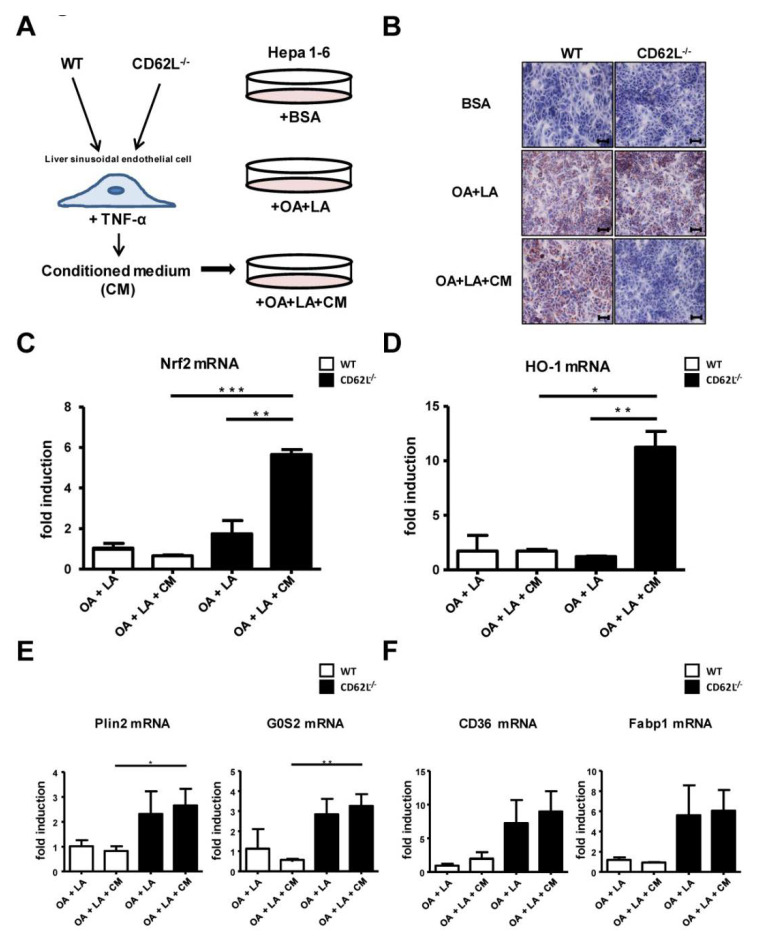
Activation of the anti-oxidative stress response and increased fat turnover in Hepa1-6 cells after stimulation with medium of activated liver sinusoidal endothelial cells (LSEC) from CD62L^−/−^ mice. (**A**) Isolated primary LSEC of WT and CD62L^−/−^ mice were activated by stimulation with bovine serum albumin (BSA) (control) or TNF-α for 24 h. Hepa1-6 cells were stimulated with linoleic acid-oleic acid-albumin (OA + LA) and additionally with conditioned medium (CM) (OA + LA + CM) of TNF-α-stimulated primary LSEC of WT and CD62L^−/−^ mice for 24 h. (**B**) Representative Oil Red O-stained tissue culture slides are depicted showing the progressive decrease of Oil Red O-staining in CD62L^−/−^ mice after stimulation with OA + LA + CM (400×). (**C**) Nrf2, (**D**) HO-1, (**E**) Plin2, G0S2, (**F**) CD36 and Fabp1 mRNA expression was analysed in the stimulated Hepa1-6 cells via RT-qPCR. For quantification values are expressed as fold induction over the mean values obtained for OA + LA treated Hepa1-6 cells (* *p* < 0.05, ** *p* < 0.01, and *** *p* < 0.001).

Next, we aimed to define the underlying mechanism responsible for less disease development and progression in CD62L^−/−^ animals. We performed in vitro experiments to assess whether loss of CD62L, especially the related differential activation of the endothelium, has a direct impact on fat metabolism in hepatocytes. Therefore, primary liver sinusoidal endothelial cells (LSEC) of WT and CD62L^−/−^ mice were stimulated with tumour necrosis factor-α (TNF-α). After activation the supernatant (conditioned medium (CM)) was used to stimulate Hepa1-6 cells with simultaneous exposure to fatty acids (oleic acid (OA) + linoleic acid (LA)) (Figure 5A). Oil Red O staining strengthened the in vivo findings as Hepa1-6 cells stimulated with CM of CD62L^−/−^ cells displayed decreased hepatocyte steatosis (Figure 5B). Decreased steatosis correlated with a significant increase in mRNA expression of typical markers of the anti-oxidative stress response, e.g., Nrf2 (Figure 5C) and HO-1 (Figure 5D) in Hepa1-6 cells after treatment with CM of CD62L^−/−^ animals. Reduced steatosis in cells stimulated with CM of CD62L^−/−^ LSEC was associated with changes in lipid metabolism shown by increased mRNA expression of Plin2, G0S2 (Figure 5E) and CD36, Fabp1 (Figure 5F) in cells stimulated with CM of CD62L^−/−^ compared to cells stimulated with WT CM highlighting a stronger fat turnover caused by CD62L deficiency.

### 3.8. Therapeutic CD62L Intervention Leads to Improved Disease Outcome in Diet-Induced Steatohepatitis

After we found ameliorated disease progression in CD62L^−/−^ animals, we next tested if this can be targeted therapeutically. We fed WT animals for 4 weeks with MCD alone and proceeded MCD treatment for additional 2 weeks combined with an anti-CD62L antibody (AB), or the respective isotype control (Figure S11).

**Figure 6:**
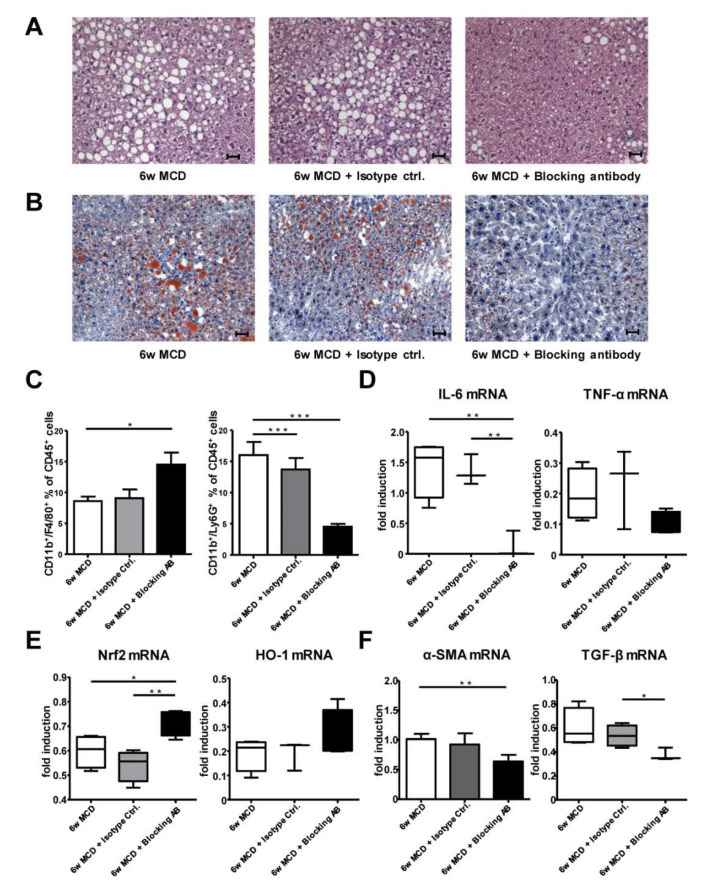
Therapeutic intervention with a CD62L blocking antibody results in dampened pro-inflammatory changes and stronger anti-oxidative stress responses in liver. (**A**) Representative H&E images or (**B**) Oil Red O stains of livers from WT mice after 6 w MCD with or without peritoneal administration of an isotype control antibody or a CD62L blocking antibody from week 4–6 after MCD feeding (400×, scale bars 50 µm). (**C**) Intrahepatic CD11b^+^/F4/80^+^ cells were analysed by FACS after 6 w MCD with or without peritoneal administration of an isotype control antibody or a CD62L blocking antibody from week 4–6 after MCD feeding. Cells were gated by FSC/SSC; duplets were excluded. Live/CD45+, then gated on CD11b^+^ and F4/80^+^ (* *p* < 0.05). Intrahepatic neutrophils were analysed by FACS in the same groups. Cells were gated by FSC/SSC; duplets were excluded. Live/CD45^+^, CD11b^+^/Ly6G^+^ were regarded as neutrophils (*** *p* < 0.001). (**D**) Hepatic IL-6 and TNF-α, (**E**) Nrf and HO-1, (**F**) α-SMA and TGF-β mRNA expression was analysed in the same groups via RT-qPCR. Values are expressed as fold induction over the mean values obtained for WT mice treated with MCD diet for 6 weeks (* *p* < 0.05, ** *p* < 0.01). All data are expressed as mean ± SEM (*n* = 4). All experiments were repeated at least twice.

MCD feed WT animals with AB treatment compared to isotype control showed significantly improved maintenance of liver architecture (H&E, Figure 6A) and less fat accumulation in hepatocytes (Oil Red O, Figure 6B). As CD62L is important for cell migration especially for Ly6G+ neutrophils, we next analysed liver immune cell infiltration. Interestingly, animals receiving the therapeutic antibody displayed an increased influx of F4/80+ monocytes compared to animals receiving MCD alone or isotype control, whereas the infiltration of Ly6G^+^ neutrophils was reduced in AB treated mice (Figure 6C). In addition, measurement of the pro-inflammatory cytokines IL-6 and TNF-α revealed that AB-treated animals show a reduced expression of IL-6 and TNF-α (Figure 6D). In contrast, these pro-inflammatory cytokines were elevated in mice receiving MCD for 6 weeks or the isotype control.

Next, we analysed the link between CD62L inhibition and the impact on the anti-oxidative stress response. RT-qPCR analysis revealed an increase in Nrf2 and HO-1 expression, while ROS production was decreased as evidenced by decreased numbers of DHE^+^ cells within liver tissue (Figure S12A,B) in AB-treated animals compared to the other groups (Figure 6E). Additionally, there was a significant increase in FoxP3 expression (Figure S12C) as a marker of Treg cells further supporting an activation of the anti-inflammatory immune response after AB intervention. Therapeutic treatment with antibody targeting CD62L significantly decreased expression of fibrosis markers as shown for α-SMA and TGF-β (Figure 6F) as well as Sirius Red positive areas after 6 weeks of treatment compared to the respective controls (Figure S13A,B).

Finally, we tested the functional relevance of these findings in vitro. We isolated primary LSEC of untreated WT mice and WT animals 24h after anti-CD62L administration. Cells were stimulated with TNF-α. The supernatant (conditioned medium (CM)) was used to stimulate Hepa1-6 cells with simultaneous exposure to fatty acids (oleic acid (OA) + linoleic acid (LA)) (Figure S14A). Oil Red O staining again revealed strong reduction in hepatocyte steatosis in Hepa1-6 cells upon stimulation with CM of cells from anti-CD62L treated WT mice (Figure S14B). The decrease in the degree of steatosis correlated with a significant increase in the expression of the anti-oxidative stress response markers Nrf2 (Figure S14C) and HO-1 (Figure S14D) in Hepa1-6 cells after stimulation with conditioned media of WT cells from anti-CD62L treated animals.

In summary, these results demonstrate that inhibiting CD62L via knockout or a blocking antibody activates the anti-inflammatory as well as the anti-oxidative stress response by modifying endothelial cell function.

## 4. Discussion

Although NAFLD is the fastest growing indication for liver transplantation in industrialized nations, the mechanisms of disease initiation, especially the involvement of cell adhesion molecule-dependent migration of different immune cell subtypes, is not well understood.

Several studies in systemic autoimmune and chronic inflammatory diseases, e.g., systemic lupus erythematosus (SLE), rheumatoid arthritis, or chronic lung diseases describe increased sCD62L serum levels [35,36,45], together with other elevated adhesion molecules, such as ICAM-1 and VCAM-1 [46,47,48]. VCAM-1 is significantly elevated in serum of NAFLD patients and it has been proposed as clinical biomarker for diagnosis of fibrosis [49]. The up-regulation of adhesion molecules is known to modulate lymphocyte migration and lymphocyte homing to sites of inflammation. Here, we found that increased sCD62L correlates with acute and chronic liver injury (Figure 1). Interestingly, patients with ulcerative colitis do not show elevated sCD62L per se. However, Vedolizumab, a blocking antibody against the cell adhesion molecule α4β7 integrin, induces CD62L shedding probably to compensate for the interruption of the α4β7 integrin-MAdCAM-1 extravasation cascade. In agreement, patients with high CD62L expression levels show low β7 integrin expression, but comparable expression patterns of AMs MAdCAM-1, ICAM-1 and VCAM-1 whose role in acute and chronic liver injury has already been demonstrated [50]. Increased expression of CD62L was further associated with clinical features of steatosis, NASH, ASH, and primary sclerosing cholangitis (PSC). CD62L expression correlated with increased intrahepatic fat accumulation in steatotis patients.

To mechanistically connect the clinical association of CD62L expression with immunological features, we performed FACS analysis revealing an increased infiltration in nearly all CD62L expressing immune cell subtypes besides of neutrophils. Kotecha et al. could show already, in 1998, which cleaved CD62L can interact with surrounding cells in areas of inflammation to promote migration through the endothelium [36]. Further, CD62L—more specifically its decreasing surface expression through shedding—is a well described marker for neutrophil activation [51,52,53]. Therefore, it is conceivable that in NASH patients CD62L expression is decreased on neutrophils upon their activation during disease initiation and progression. This theory is supported by a significant decrease in infiltrating intrahepatic neutrophils in CD62L^−/−^ animals after treatment with steatohepatitis-inducing diets (Figure 3) and results observing a beneficial effect of blocking CD62L in a murine liver reperfusion model showing diminished neutrophil activity [37].

Earlier studies established the beneficial effects of blocking the interaction between cell adhesion molecules and their endothelial receptors in different diseases. In chronic inflammatory bowel disease, this inhibition already reached clinical practice by suppressing the pro-inflammatory immune response and immune cell infiltration [54,55]. Here, loss of CD62L resulted in less accumulation of CD11b^+^/Ly6G^+^ neutrophils while CD62L^−/−^ mice showed increased numbers of macrophages and CD4^+^ T cells. Both cell types tend to exhibit a strong anti-inflammatory phenotype by high expression of either markers typically expressed by M2 macrophages (IL-10), the T cell inhibitory factor and Treg-associated molecule CTLA-4, and Treg markers, such as CD25 and FoxP3 (Figure 3). While at the same time, the expression of markers typically known to promote a pro-inflammatory cytokine environment are downregulated in these mice (IL-6, IL-1β) [56].

In line with dropping neutrophil numbers, others described that inhibiting neutrophils positively influences the development of chronic hepatitis B virus (HBV) induced liver injury [57]. Additionally, hepatitis C virus (HCV) treated patients showed decreased numbers of neutrophils [58]. In the context of NASH, it could directly link the accumulation and activation of neutrophils with severe outcome in a high fat steatohepatitis mouse model [59].

These studies, together with our observations showing decreased numbers of neutrophils in CD62L^−/−^ livers after feeding steatohepatitis-inducing diets, suggest a key role of these cells in promoting CD62L-dependent initiation from steatosis to steatohepatitis. Strong accumulation of T cells expressing anti-inflammatory markers further assist to dampen disease manifestation. In line, a new subpopulation of CD62L^+^ neutrophils was primarily described having the ability to inhibit pro-inflammatory T cell responses by the release of hydrogen peroxide [60].

Unexpected, besides differences in immune cell composition, the loss of CD62L was not associated with a compensatory regulation of other cell adhesion molecules such as VCAM-1 or ICAM-1. Endothelial dysfunction is a pathophysiological hallmark of NAFLD development [61]. The expression of adhesion molecules is regulated by pro-inflammatory cytokines distributed after endothelial activation at the site of inflammation [62,63]. Therefore, we hypothesize, that the lack of CD62L leads to reduced CD62L-mediated immune cell/endothelial cell interaction resulting in less endothelial activation, finally protecting CD62L^−/−^ mice from initiation and progression of steatohepatitis.

Milder disease progression in CD62L^−/−^ mice is associated with an ameliorated oxidative stress response. Conversely, strong activation of the anti-oxidative stress response can be observed in these mice finally leading to less accumulation of intrahepatic but also visceral and subcutaneous fat. In vitro experiments suggest a direct functional connection between CD62L dependent endothelial activation, steatosis and initiation towards steatohepatitis mediated via an anti-oxidative stress response and lipid turnover in hepatocytes.

The important role of oxidative stress caused by toxic lipids and the anti-oxidative stress response during insulin resistance, metabolic disorders, and steatohepatitis development is widely accepted [64]. Previously we have shown that Nrf2 over-activation in hepatocytes after Keap1 deletion resulted in diminished hepatocyte steatosis and progression to steatohepatitis [65]. Recent studies demonstrated a direct protective effect of HO-1 on insulin resistance [66]. Others discovered that FoxP3 an essential transcription factor in Tregs directly induces HO-1 expression [67]. These findings were supported in CD62L^−/−^ livers showing a massive increase in CD25^+^FoxP3^+^ cells together with strong HO-1 expression. In vitro experiments further show an intense activation of HO-1 in hepatocytes stimulated with fatty acids together with medium of previously activated CD62L^−/−^ endothelial cells (Figure 5).

CD62L^−/−^ animals display less extracellular matrix accumulation under steatohepatitis-inducing diet feeding. In a recent study, it was shown that chronic liver injury leads to recruitment and differentiation of bone marrow derived LSEC progenitors [68]. Under physiological conditions, these cells are described to maintain the quiescent state of hepatic stellate cells (HSCs). If these cells fail to fully differentiate because of chronic liver damage, they lose the ability to suppress HSC activity and promote hepatic fibrosis. In the course of the mild disease progression in CD62L^−/−^ mice less endothelial cell activation may in part be responsible for decreased matrix deposition. Supporting this hypothesis another study has demonstrated that HSC activation and proliferation is further induced by neutrophils, which are an important cell type associated with enhanced liver fibrosis in NASH patients [69]. Already in obese patients, the neutrophil numbers are significantly increased [53]. In agreement, the neutrophil-to-lymphocyte ratio was found to be a non-invasive marker associated with NASH and NASH-fibrosis [70].

Several groups investigated the beneficial effect of inhibiting CD62L activation via antibody. In 2017, the first study was published using a CD62L antibody using a 12-week HFD model before hepatic ischemia reperfusion injury [71]. Thereby, the authors could show that animals receiving the blocking antibody had less liver necrosis and decreased IL-6 levels. The authors suggested that ameliorated disease progression is due to diminished CD62L-mediated trafficking of CD8+ cells leading to less hepatocellular injury. Upon blocking CD62L during MCD treatment, we found decreased infiltration of neutrophils together with lower levels of IL-6 and TNF-α. Additionally, antibody administration triggered a stronger anti-oxidative stress response and decreased extracellular matrix deposition.

Our study clearly demonstrated that CD62L expression correlated with disease activity of steatohepatitis in mice and men, while the lack of CD62L was associated with beneficial effects in preventing steatosis and fibrosis. Since NAFLD and NASH are critical risk factors for HCC development, it is tempting to speculate that targeting CD62L might be effective in preventing hepatocarcinogenesis. Although we have not tested long-term effects of MCD and HFD in CD62L^−/−^ mice, the human protein atlas classifies CD62L as a general cancer-related gene with low cancer specificity. Increased expression is found in glioma, lung cancer, colorectal cancer, liver cancer, urothelial cancer, breast cancer, cervical cancer, endometrial cancer, and lymphoma (https://www.proteinatlas.org/ENSG00000188404-SELL/pathology). Therefore, it will be interesting to investigate nutritional-induced HCC formation in CD62L^−/−^ mice in future studies.

Taken together, our results show that the loss or antibody-mediated blockade of CD62L protects against initiation and progression of diet-induced steatohepatitis. Decreased disease progression could be functionally linked to alterations in pro-inflammatory immune cell infiltration, together with diminished endothelial activation and a strong activation of the anti-oxidative stress response. Thus, we defined CD62L as a promising novel therapeutic NAFLD target.

## Figures and Tables

**Table 1:** Primary antibodies for immunofluorescence staining.

Immunoreactivity	Dilution	Species	Manufacturer	Cat. No.
Ly6G	1:100	Rat	BD Pharmingen, Heidelberg, Germany	550291
F4/80	1:100	Rat	Biorad, Hercules, CA USA	MCA497GA
DHE	1:500	-	Invitrogen, Carlsbad, CA, USA	D23107
VCAM-1	1:100	Rat	BD Pharmingen	553329
ICAM-1	1:100	Rat	eBioscience, Santa Clara, CA, USA	14-0541-81

Abbreviations used are DHE, Dihydroethidium (hydroethidine); ICAM-1, intercellular adhesion molecule 1; Ly6G, lymphocyte antigen 6 complex, VCAM-1, vascular cell adhesion protein 1.

**Table 2:** Primers used in this study.

Gene	Species	Sequence
GAPDH	Mouse	for: 5′-TGT TGA AGT CAC AGG AGA CAA CCT-3′ rev: 5′-AAC CTG CCA AGT ATG ATG ACA TCA-3′
TGF-β1	Mouse	for: 5′-ATA CGC CTG AGT GGC TGT CT -3′ rev: 5′-TCA TGG ATG GTG CCC AGG TC-3′
Nrf2	Mouse	for: 5′-CCG AGA TAT ACG CAG GAG AGG TA-3′ rev: 5′-GCT CGA CAA TGT TCT CCA GCT T-3′
HO-1	Mouse	for: 5′-TGG TGG CCC ACG CAT ATA C-3′ rev: 5′-CAT GGC CTT CTG TGC AAT CTT-3′
IL-10	Mouse	for: 5′-GAT GCC CCA GGC AGA GAA-3′ rev: 5′-CAC CCA GGG AAT TCA AAT GC-3′
FoxP3	Mouse	for: 5′-GGC AAA TGG AGT CTG CAA GTG-3′ rev: 5′-CAG GAG ATG ATC TGC TTG GCA-3′
TNF-α	Mouse	for: 5′-AGC TCG TAG CAA ACC ACC AA-3′ rev: 5′-GAG AAC CTG GGA GTA GAC AAG G-3′
IL-1β	Mouse	for: 5′-GGA GAA CCA AGC AAC GAC AAA-3′ rev: 5′-GGG TGT GCC GTC TTT CAT TAC-3′
ADAM-17	Mouse	for: 5′-CAC AAA ACT TGA GAG TCG TGG T-3′ rev: 5′-GCT AGA ACC CTA GAG TCA GGC-3′
IL-6	Mouse	for: 5′-CTG CAA GAG ACT TCC ATC CAG-3′ rev: 5′-AGT GGT ATA GAC AGG TCT GTT GG-3′
Plin2	Mouse	for: 5′-GTC CAC CTG ATT GAA TTC GC-3′ rev: 5′-CGA TGT GCT CAA CAC AGT G-3′
G0S2	Mouse	for: 5′-ACT GCA CCC TAG GCC CAG-3′ rev: 5′-GTC TCA ACT AGG CCG AGC AC-3′
CD36	Mouse	for: 5′-CAA ATG CAA AGA AGG AAA GCC-3′ rev: 5′-AAT GGT CCC AGT CTC ATT TAG C-3′
Fabp1	Mouse	for: 5′-GCA GAG CCA GGA GAA CTT TG-3′ rev: 5′-GGG TCC ATA GGT GAT GGT GAG- 3′
β-Actin	Human	for: 5′-TCC ATC ATG AAG TGT GAC GT-3′ reverse: TAC TCC TGC TTG CTG ATC CAC-3′
RPLP0	Human	for: 5′-ACT GTG CCA GCC CAG AAC A-3′ rev: 5′-AGC CTG GAA AAA GGA GGT CTT C-3′
CD62L	Human	for: 5′-GGA CTG CGT GGA GAT CTA TAT CAA-3′ rev: 5′-TGG CAG GCG TCA TCG TT-3′
β_7_-Integrin	Human	for: 5′-GGA CTC CAG CAA CGT GGT ACA-3′ rev: 5′-TCA CGG TGG AAG ACA GGC TAT-3′
MAdCAM-1	Human	for: 5′-TGA GTG GCC AGC CTT TCC-3′ rev: 5′-CCC TGA CCA GTT CTC AAC TTG AA-3′
ICAM-1	Human	for: 5′-TGG CCC TCC ATA GAC ATG TGT-3′ rev: 5′-TGG CAT CCG TCA GGA AGT G-3′
VCAM-1	Human	for: 5′-CAA AGG CAG AGT ACG CAA ACA C-3′ rev: 5′-GCT GAC CAA GAC GGT TGT ATC TC-3′

Abbreviations used: ADAM-17, a disintegrin and metalloproteinase domain 17; CD36, cluster of differentiation 36; CD62L, Selectin L; Fabp1, fatty acid-binding protein 1; for, forward primer; FoxP3, Forkhead box P3; GAPDH, Glyceraldehyde 3-phosphate dehydrogenase; G0S2, Go(G1 switch gene 2; HO-1, heme oxygenase 1; ICAM-1, intercellular adhesion molecule 1; IL-1β, interleukin-1β; IL-10, interleukin-10; IL-6, interleukin-6; MAdCAM-1, Mucosal vascular addressin cell adhesion molecule; Nrf2, NEF2-related factor 2; Plin2, Perlipin 2; rev, reverse primer, RPLP0, ribosomal phosphoprotein large P0; TGF-β1, transforming growth factor-β1; TNF-α, tumour necrosis factor-α; VCAM-1, vascular cell adhesion protein 1.

**Table 3:** Antibody panels used for flow cytometric analysis (FACS) analysis.

Cross-Reactivity (Panel)	Antibody	Conjugate	Manufacturer	Cat. No.
Mouse (1)	CD45	APC-Cy7	BD Pharmingen, Heidelberg, Germany	557659
	F4/80	APC	eBioscience, Santa Clara, CA, USA	47-4801
	CD11b	PE	eBioscience	12-0112-82
	CD11c	PE-Cy7	eBioscience	25-0114-81
	Ly6G/Ly6C	PerCP-Cy5.5	Biolegend, San Diego, CA, USA	127615
	Ly6G	FITC	eBioscience	17-5931-81
	Hoechst 33258	-	Sigma Aldrich, Merck, Taufkirchen, Germany	B2883
Mouse (2)	CD45	APC-Cy7	BD Pharmingen	557659
	CD3	PE-Cy7	Biolegend	100219
	CD4	FITC	eBioscience	11-0042-82
	CTLA4	PE	eBioscience	12-1522-81
	CD8	PerCP-Cy5.5	BD Pharmingen	551162
	CD25	PE	eBioscience	12-0025-81
	FoxP3	APC	eBioscience	17-5773-80
	Hoechst 33258	-	Sigma Aldrich	B2883
Human (1)	CD45	BV510	BD Pharmingen	563204
	CD4	APC-Cy7	BD Pharmingen	561839
	CD8	AF700	BD Pharmingen	557945
	CD62L	PE-Cy7	BD Pharmingen	565535
	CD66b	APC	eBioscience	17-0666-42
	CD14	VioGreen	Milteny Biotech, Bergisch Gladbach, Germany	130-096-875
	CD68	PE-eFluor610	eBioscience	61-0689-41
	Hoechst 33258	-	Sigma Aldrich	B2883

Abbreviations used are APC, Allophycocyanin; BV510, brilliant violet 510; CD, cluster of differentiation, FITC, Fluorescein isothiocyanate; Ly6G/Ly6C, lymphocyte antigen 6 complex, PE, Phycoerythrin.

**Table 4:** Composition of buffers used for primary liver sinusoid endothelial cell isolation.

Chemical	SC1	SC2	GBSS-A	GBSS-B
NaCl	8000 mg/L	8000 mg/L	-	8000 mg/L
KCl	400 mg/L	400 mg/L	370 mg/L	370 mg/L
NaH_2_PO_4_ × H_2_O	88.17 mg/L	88.17 mg/L	-	-
Na_2_HPO_4_	120.45 mg/L	120.45 mg/L	59.6 mg/L	59.6 mg/L
HEPES	2380 mg/L	2380 mg/L	-	-
NaHCO_3_	350 mg/L	350 mg/L	227 mg/L	227 mg/L
EGTA	190 mg/L	-	-	-
Glucose	900 mg/L	-	991 mg/L	991 mg/L
CaCl_2_ × 2H_2_O	-	560 mg/L	225 mg/L	225 mg/L
MgCl_2_ × 6H2O	-	-	210 mg/L	210 mg/L
MgSO_4_ × 7H2O	-	-	70 mg/L	70 mg/L
KH_2_PO_4_	-	-	30 mg/L	30 mg/L

Abbreviations used are: EGTA, ethylene glycol bis(beta-aminoethyl ether)-N,N,N′,N′-tetraacetic acid; GBSS, Gey’s balanced salt solution; HEPES, 4-(2-hydroxyethyl)-1-piperazineethanesulfonic acid.

## Supplementary Materials

The following are available online at https://www.mdpi.com/2073-4409/9/5/1106/s1, Figure S1: Adhesion molecule expression in steatosis patients, Figure S2: Longitudinal µCT analysis of wild type mice, Figure S3: Longitudinal µCT analysis of CD62L−/− mice, Figure S4: NAFLD activity score staining, Figure S5: Fat accumulation in wild type and CD62L−/− mice, Figure S6: Gating strategy for flow cytometric (FACS) analysis, Figure S7: Oxidative stress response after treatment with steatosis-inducing diets in wild type and CD62L−/− mice, Figure S8: Regulation of compensatory adhesion molecules, Figure S9: Therapeutic CD62L intervention, Figure S10: Oxidative stress response after therapeutic CD62L intervention, Figure S11: Fibrosis initiation after therapeutic CD62L intervention; Figure S12: Activation of the anti-oxidative stress response in Hepa1-6 cells after stimulation with activated sinusoidal endothelial cells from wild type animals after therapeutic CD62L intervention, Table S1: Baseline patient demographic and disease characteristics for serum ELIS analysis, quantitative real time PCR analysis, and cytometric analysis; Representative health reports of animals used in this study is also included.
