# Supplementary material for: L-Selectin/CD62L Is a Key Driver of Non-Alcoholic Steatohepatitis in Mice and Men
Source: Cells. 2020 Apr 29;9(5):1106. doi: 10.3390/cells9051106 (PMC7290433; doi:10.3390/cells9051106)
Supplement: Supplementary file 1 [file cells-09-01106-s001.zip › cells-779835 non pub mat.pdf]

Appendix A: Representative health reports of animals used in this study

Institute for Laboratory Animal Science
University Hospital RWTH Aachen
Pauwelsstr. 30, 52074 Aachen
Germany

PD Dr. phil. nat. Julia Steitz
Fr. Katharina Pietsch
Tel.: +49 241 80 - 88603
Fax: +49 241 80 - 82462
Email:
jsteitz@ukaachen.de
kpietsch@ukaachen.de

Health Report **FB-L-MIBI-013-04** Date of issue: 29-Sep-16

Hygienic unit:	**Barrier yellow, Floor 55, Room 4**	U-Nr.:	**259,260/2016**
Opening:	August 2014	Animals tested:	Sentinels - CD-1
Species within the unit:	Mice	Age at time of control	4 month
Owner:	**Dr. rer nat. Angela Schippers**<br>Klinik für Kinder- und Jugendmedizin	Control:	**3/4 in 2016**

VIRUSES	Test frequency	Latest test date	Latest results	Historical results ≤ 18 months	Test method	Test laboratory
Lymphocytic choriomeningits virus (LCM)	annually	10-Nov-15	0/2	0/2	IFA	BioDoc
Minute virus of mice (MVM)	quarterly	03-Aug-16	0/2	0/12	IFA	BioDoc
Mouse adenovirus type 1 (FL)	annually	10-Nov-15	0/2	0/2	IFA	BioDoc
Mouse adenovirus type 2 (K87)	annually	10-Nov-15	0/2	0/2	IFA	BioDoc
Mouse hepatitis virus (MHV)	quarterly	03-Aug-16	0/2	0/12	IFA	BioDoc
Mouse parvovirus (MPV)	quarterly	03-Aug-16	0/2	0/12	IFA	BioDoc
Mouse rotavirus (EDIM)	quarterly	03-Aug-16	0/2	0/12	IFA	BioDoc
Mousepox (ectromelia) virus	annually	10-Nov-15	0/2	0/2	IFA	BioDoc
Murine norovirus	quarterly	03-Aug-16	0/2	0/12	IFA	BioDoc
Pneumonia virus of mice (PVM)	annually	10-Nov-15	0/2	0/2	IFA	BioDoc
Reovirus type 3	annually	10-Nov-15	0/2	0/2	IFA	BioDoc
Sendai virus	annually	10-Nov-15	0/2	0/2	IFA	BioDoc
Theiler's murine encephalomyelitis virus (TMEV)	quarterly	03-Aug-16	0/2	0/12	IFA	BioDoc

BACTERIA / MYCOPLASMA / FUNGI	Test frequency	Latest test date	Latest results	Historical results ≤ 18 months	Test method	Test laboratory
Citrobacter rodentium	quarterly	03-Aug-16	0/2	0/12	CULT	ILAS
Clostridium piliformis (Tyzzer's disease)	annually	10-Nov-15	0/2	0/2	IFA	BioDoc
Corynebacterium kutscheri	quarterly	03-Aug-16	0/2	0/12	CULT	ILAS
Helicobacter spp.	quarterly	03-Aug-16	0/2	0/12	PCR	BioDoc
Mycoplasma pulmonis	annually	10-Nov-15	0/2	0/2	IFA	BioDoc
Pasteurella pneumotropica	quarterly	03-Aug-16	0/2	0/12	IFA	BioDoc
Pasteurella pneumotropica	quarterly	03-Aug-16	0/2	0/12	CULT	ILAS
Salmonella spp.	quarterly	03-Aug-16	0/2	0/12	CULT	ILAS
ß-hemolytic Streptococci (not group D)	quarterly	03-Aug-16	0/2	0/12	CULT	ILAS
Streptobacillus moniliformis	quarterly	03-Aug-16	0/2	0/12	CULT	ILAS
Streptococcus pneumoniae	quarterly	03-Aug-16	0/2	0/12	CULT	ILAS

PARASITES	Test frequency	Latest test date	Latest results	Historical results ≤ 18 months	Test method	Test laboratory
Ectoparasites	quarterly	03-Aug-16	0/2	0/12	MICR	ILAS
Endoparasites	quarterly	03-Aug-16	0/2	0/12	Flotation/Tape Test	ILAS

Pathological lesions observed	Test frequency	Latest test date	Latest results	Historical results ≤ 18 months	Test method	Test laboratory
	quarterly	03-Aug-16	0/2	0/12	PATH	ILAS

BioDoc: Prof. Dr. M. Mähler, Hannover, Germany
ILAS: Inst. f. Laboratory Animal Science, University Hospital RWTH Aachen
*Trichomonas spp.

Data are expressed as number positive animals/number examined.

Screening samples: animals are tested at least quarterly. Necropsy, pathology, number of samples and methods comply with the "FELASA recommendations for the health monitoring of mouse, rat, hamster, guinea pig and rabbit colonies in breeding and experimental units." (Lab Anim. 2014 Mar 4.)

Abbreviations used in this report:
ELISA = enzyme-linked immunosorbent assay, MICR = microscopy, IFA = Immunfluorescence assay, CULT = culture, PATH = gross pathology, PCR = polymerase chain reaction, HIST = histopathology, NT = not tested

Appendix A: Representative health reports of animals used in this study

Institute for Laboratory Animal Science
University Hospital RWTH Aachen
Pauwelsstr. 30, 52074 Aachen
Germany

iLAS

PD Dr. phil. nat. Julia Steitz
Fr. K. Pietsch
Tel.: +49 241 80 - 88603
Fax: +49 241 80 - 82462
Email:
jsteitz@ukaachen.de
kpietsch@ukaachen.de

Health Report

FB-L-MIBI-013-04 | Date of issue: 29-Sep-16

Hygienic unit:	**Barrier Red, Floor 53**	U-Nr.:	**246-256/16**
Opening:	December 2009	Animals tested:	Sentinels CD-1
Species within the unit:	Mice, genetically modified, breeding	Age at time of control:	7 months
Owner:	**Dr. rer nat Angela Schippers**	Control:	**3/4 in 2016**
	**Klinik für Kinder- und Jugendmedizin**		

**VIRUSES**	**Test frequency**	**Latest test date**	**Latest results**	**Historical results ≤ 18 months**	**Test method**	**Test laboratory**
Lymphocytic choriomeningits virus (LCM)	annually	03-Nov-15	0/13	0/13	IFA	BioDoc
Minute virus of mice (MVM)	quarterly	05-Jul-16	0/14	0/80	IFA	BioDoc
Mouse adenovirus type 1 (FL)	annually	03-Nov-15	0/13	0/13	IFA	BioDoc
Mouse adenovirus type 2 (K87)	annually	03-Nov-15	0/13	0/13	IFA	BioDoc
Mouse hepatitis virus (MHV)	quarterly	05-Jul-16	0/14	0/80	IFA	BioDoc
Mouse parvovirus (MPV)	quarterly	05-Jul-16	0/14	0/80	IFA	BioDoc
Mouse rotavirus (EDIM)	quarterly	05-Jul-16	0/14	0/80	IFA	BioDoc
Mousepox (ectromelia) virus	annually	03-Nov-15	0/13	0/13	IFA	BioDoc
Murine norovirus	quarterly	05-Jul-16	0/14	0/80	IFA	BioDoc
Pneumonia virus of mice (PVM)	annually	03-Nov-15	0/13	0/13	IFA	BioDoc
Reovirus type 3	annually	03-Nov-15	0/13	0/13	IFA	BioDoc
Sendai virus	annually	03-Nov-15	0/13	0/13	IFA	BioDoc
Theiler's murine encephalomyelitis virus (TMEV)	quarterly	05-Jul-16	0/14	0/80	IFA	BioDoc

**BACTERIA / MYCOPLASMA / FUNGI**	**Test frequency**	**Latest test date**	**Latest results**	**Historical results ≤ 18 months**	**Test method**	**Test laboratory**
Citrobacter rodentium	quarterly	05-Jul-16	0/14	0/80	CULT	ILAS
Clostridium piliformis (Tyzzer's disease)	annually	03-Nov-15	0/13	0/13	IFA	BioDoc
Corynebacterium kutscheri	quarterly	05-Jul-16	0/14	0/80	CULT	ILAS
Helicobacter spp.	quarterly	05-Jul-16	0/14	0/80	PCR	BioDoc
Mycoplasma pulmonis	annually	03-Nov-15	0/13	0/13	IFA	BioDoc
Pasteurella pneumotropica	quarterly	05-Jul-16	0/14	0/80	IFA	BioDoc
Pasteurella pneumotropica	quarterly	05-Jul-16	0/14	0/80	CULT	ILAS
Salmonella spp.	quarterly	05-Jul-16	0/14	0/80	CULT	ILAS
ß-hemolytic Streptococci (not group D)	quarterly	05-Jul-16	0/14	0/80	CULT	ILAS
Streptobacillus moniliformis	quarterly	05-Jul-16	0/14	0/80	CULT	ILAS
Streptococcus pneumoniae	quarterly	05-Jul-16	0/14	0/80	CULT	ILAS

**PARASITES**	**Test frequency**	**Latest test date**	**Latest results**	**Historical results ≤ 18 months**	**Test method**	**Test laboratory**
Ectoparasites	quarterly	05-Jul-16	0/14	0/80	MICR	ILAS
Endoparasites	quarterly	05-Jul-16	0/14	0/80	Flotation/Tape Test	ILAS

**Pathological lesions observed**	**Test frequency**	**Latest test date**	**Latest results**	**Historical results ≤ 18 months**	**Test method**	**Test laboratory**
	quarterly	05-Jul-16	0/14	0/80	PATH	ILAS

BioDoc: Prof. Dr. M. Mähler, Hannover, Germany
ILAS: Inst. f. Laboratory Animal Science, University Hospital RWTH Aachen

Data are expressed as number positive animals/number examined.

Screening samples: animals are tested at least quarterly. Necropsy, pathology, number of samples and methods comply with the "FELASA recommendations for the health monitoring of mouse, rat, hamster, guinea pig and rabbit colonies in breeding and experimental units." (Lab Anim. 2014 Mar 4.)

Abbreviations used in this report:
ELISA = enzyme-linked immunosorbent assay, MICR = microscopy, IFA = Immunfluorescence assay, CULT = culture, PATH = gross pathology, PCR = polymerase chain reaction, HIST = histopathology, NT = not tested

FB-L-MIBI-013-04.xls | Stand: 01.10.2014
